# Role of *BRCA2 *mutation status on overall survival among breast cancer patients from Sardinia

**DOI:** 10.1186/1471-2407-9-62

**Published:** 2009-02-20

**Authors:** Mario Budroni, Rosaria Cesaraccio, Vincenzo Coviello, Ornelia Sechi, Daniela Pirino, Antonio Cossu, Francesco Tanda, Marina Pisano, Grazia Palomba, Giuseppe Palmieri

**Affiliations:** 1 Registro Tumori di Sassari, ASL 1, Sassari, Italy; 2 Dipartimento Prevenzione, ASL BA1, Bari, Italy; 3 Istituto di Anatomia Patologica, Università di Sassari, Sassari, Italy; 4 Unità Genetica dei Tumori, Istituto di Chimica Biomolecolare-CNR, Sassari, Italy

## Abstract

**Background:**

Germline mutations in *BRCA1 *or *BRCA2 *genes have been demonstrated to increase the risk of developing breast cancer. Conversely, the impact of *BRCA *mutations on prognosis and survival of breast cancer patients is still debated. In this study, we investigated the role of such mutations on breast cancer-specific survival among patients from North Sardinia.

**Methods:**

Among incident cases during the period 1997–2002, a total of 512 breast cancer patients gave their consent to undergo *BRCA *mutation screening by DHPLC analysis and automated DNA sequencing. The Hakulinen, Kaplan-Meier, and Cox regression methods were used for both relative survival assessment and statistical analysis.

**Results:**

In our series, patients carrying a germline mutation in coding regions and splice boundaries of *BRCA1 *and *BRCA2 *genes were 48/512 (9%). Effect on overall survival was evaluated taking into consideration *BRCA2 *carriers, who represented the vast majority (44/48; 92%) of mutation-positive patients. A lower breast cancer-specific overall survival rate was observed in *BRCA2 *mutation carriers after the first two years from diagnosis. However, survival rates were similar in both groups after five years from diagnosis. No significant difference was found for age of onset, disease stage, and primary tumour histopathology between the two subsets.

**Conclusion:**

In Sardinian breast cancer population, *BRCA2 *was the most affected gene and the effects of *BRCA2 *germline mutations on patients' survival were demonstrated to vary within the first two years from diagnosis. After a longer follow-up observation, breast cancer-specific rates of death were instead similar for *BRCA2 *mutation carriers and non-carriers.

## Background

The breast cancer is a complex disease with high biological heterogeneity and wide spectrum of responsiveness to different treatments. The well-established prognostic factors currently used into the management of patients with breast carcinoma include the disease stage (which takes into account tumour size, axillary lymph node involvement, and distant tumour dissemination) as well as the histological type, the degree of differentiation (tumour grade), the proliferation index, and the receptor status [estrogen receptor (ER), progesterone receptor (PR), and, recently, HER2] of the primary tumours [1]. Among them, the expression levels of hormone receptors seem to better predict the breast cancer response to different therapeutic strategies. More in general, the assessment of some molecular mechanisms responsible for the mammary tumourigenesis and studies on molecular profiling allowed to identify several biomarkers which may be helpful to pathologically classify breast cancer lesions into subtypes with different prognostic and clinico-pathologic behaviours [2,3].

Mutations in the *BRCA1 *and *BRCA2 *tumour suppressor genes have been associated with the breast cancer risk among families with strong recurrence of the disease [4-9]. Vast majority of studies has shown a highly increased risk of developing breast cancer in *BRCA1 *or *BRCA2 *mutation carriers [4-7] with also a greater incidence of a second contra-lateral tumour [4,5,10]. However, majority of breast cancers occur sporadically in individuals with little or no family history, for whom no clear role of the mutations in *BRCA *genes has emerged. Overall, *BRCA *mutations are responsible for 30–60% of the hereditary cases and have a prevalence of about 5% in the general population and about 25% in the families with history of breast cancer [9].

Analogously, several studies have investigated the possible effects of *BRCA *mutations on clinical and pathologic characteristics of breast cancer as well as on prognosis and survival rates of the patients, but the results were inconclusive. Some of these studies have demonstrated that *BRCA1 *mutation carriers develop cancer with a high proliferation index and low expression of estrogen receptors [11,12]. Moreover, a higher proliferation index has been reported in all breast cancer sporadic cases carrying a *BRCA *germline mutation (regardless of the gene involved) in comparison to the patients with wild-type *BRCA *[13-15]. Conversely, other authors observed no difference in histological tumour features among *BRCA2*-positive familial cases and sporadic cases [16]. Regarding the relationship with the survival, *BRCA1 *mutation carriers showed either a poor prognosis in patients with negative lymph nodes [17] or a worse outcome in comparison with *BRCA2*-positive cases [18,19]. Other investigators did not find any significant survival difference in *BRCA *mutation carriers compared with non-carrier cases [20,21]; moreover, breast cancer specific mortality rates have been found similar for *BRCA *mutation carriers and non-carriers in Jewish population [22]. Nevertheless, a better assessment of the role on survival and prognosis could be also important for women with a *BRCA *mutation who face a decision between preventive surgery and intensive surveillance.

In Sardinia, whose population is genetically homogeneous due to the fact that it is relatively isolated and with high rate of inbreeding, the contribution of *BRCA *mutations to the population incidence of breast cancer has been evaluated by our group in recent past years [23-25]. Three deleterious *BRCA *germline mutations have been observed in about 15% families and in about 3% non-familial breast cancer patients from North Sardinia (*BRCA2 *mutations were the most prevalent *BRCA *sequence variations and a single variant, *BRCA2-8765delAG*, was the most recurrent mutation with a founder effect in our population) [23-25]. In North Sardinia, breast cancer represents the principal death-causing malignancy, with an incidence rate quite comparable with that observed in Western countries (standardized rate, 95 per 100.000 inhabitants per year); the median age of onset for breast cancer among Sardinian women is 65 years [26]. Based on the existence of an official cancer registry, which has recorded all malignancies diagnosed into the population of the province of Sassari from 1992 to 2003 [26], the objective of the present study was to investigate the relationship between the occurrence of *BRCA *mutations and the main standardized prognostic factors as well as the overall survival rates among breast cancer patients from North Sardinia. Specifically, we compared survival rates between *BRCA2 *mutation carriers and non-carriers while adjusting for demographic and clinically-recognized prognostic factors in a homogenous group of Sardinian breast cancer patients.

## Methods

### Patients' selection

Among 1,835 incident cases during the period 1997–2002 [with 140 (8%) tumour-specific deaths], we selected all consecutive patients with histologically-proven diagnosis of malignant breast cancer (regardless of factors which may influence prognosis: age, family history, disease stage, or type of treatment). Among them, 512 patients gave their consent to undergo genetic analysis for detection of *BRCA *mutations on germline DNA from peripheral blood. For such cases, the collected information included the disease stage at diagnosis, the expression levels of estrogen and progesterone receptors, and the occurrence of a second cancer. All information have been verified through analysis of the hospital records; all cancer diagnoses were confirmed by pathology reports.

The study was reviewed and approved by the ethical review boards of both Institutions (University of Sassari and A.S.L.1 of Sassari).

### Mutation screening

For the *BRCA1 *and *BRCA2 *genetic testing, all patients were informed about the aims and limits of the mutation analysis and blood samples were collected after obtaining a patient's written consent (in any case, documentation of counselling was carefully evaluated prior to genetic testing). As previously described [23,24], genomic DNA samples were screened for mutations in *BRCA1 *and *BRCA2 *genes by a sequential combination of denaturing high-performance liquid chromatography (DHPLC) analysis and sequencing approach using an automated fluorescence-cycle sequencer (ABIPRISM 3100, Applied Biosystems, Foster City, CA).

### Statistical analysis

The following variables and categories were defined and included in our analyses: pathological primary tumor size (pT), pathological nodal status (pN), presence of distant metastases (M), estrogen and progesteron receptor (ER and PR, respectively) status, age at diagnosis, and overall survival (calculated starting from the time of diagnosis to the date of death or to the end of our follow-up observation on December 31, 2004). Receptor status was not known in a fraction (about 30%) of the patients included into the study.

The general mortality data were provided from official regional sources, and in some cases were drawn from the municipality rosters. The death probability was calculated on the mortality rate basis and expressed as the probability that an individual has, at beginning of the age class considered, to die before going to the next age class. The formula from life-table that assume a constant mortality rate within a given period was applied [27]. In this case, the age class was equal to one year, as required by the Hakulinen method for calculation of relative survival [28].

The five-year relative survival figures were also computed following the Hakulinen method [28]; the 95% confidence limits were calculated using the "*eurocare *" confidence interval algorithm [29]. For the comparison of survival probabilities within the various subgroups, the cumulative relative survival adjusted for age was estimated using the technique proposed by Brenner and colleagues [29].

The role of familiarity and *BRCA *status as genetic marker in cause-specific survival was investigated by Kaplan-Meier and Cox regression methods. All tests were computed by Stata Software.

## Results

Among the 512 breast cancer patients who gave their consent to participate to the study, 103 (20%) had a family history of breast cancer. Cases were classified as familial when at least three affected members (considering first- and second-degree relatives) were diagnosed with breast cancer.

Mutation analysis for all coding regions and splice boundaries of *BRCA1 *and *BRCA2 *genes was performed as previously described [24]. Briefly, germline DNA from breast cancer patients was screened by DHPLC analysis; all PCR products presenting an abnormal denaturing profile in comparison to the normal controls were sequenced using an automated approach. Taking into consideration the 103 familial cases, 2 (2%) and 20 (19%) presented a germline mutation in *BRCA1 *and *BRCA2 *genes, respectively. Among the remaining 409 patients classified as sporadic cases, 2 (0.5%) and 24 (6%) were found to carry *BRCA1 *and *BRCA2 *mutations, respectively. Overall, patients carrying a *BRCA *mutation were 48/512 (9%). In particular, *BRCA1 *mutations were detected in only 4/48 (8%) carriers, while *BRCA2 *mutations were identified in vast majority of them (44/48; 92%), with the *BRCA2-8765delAG *variant acting as a founder mutation [23-25]. Taking into account such a high preponderance of germline mutations, only *BRCA2*-positive cases were considered for statistical correlations in our series.

The age of breast cancer onset was evaluated on the basis of the mutation status; 23/44 (52%) *BRCA2 *mutation positive and 188/464 (41%) *BRCA2 *mutation negative patients were 50 years or younger at the time of diagnosis (Table 1). Although the average age at diagnosis was younger in patients carrying *BRCA2 *mutations [23/44 (52%) ≤ 50 years *vs. *21/44 (48%) > 50 years] than in cases with no detectable mutation [188/464 (41%) ≤ 50 years *vs. *276/464 (59%) > 50 years], such a difference was not statistically significant. Using Pearson's Chi-Squared test, the occurrence of a *BRCA2 *mutation was evaluated for association with several pathological parameters: pT, pN, M, or, when available, ER and PR. As shown in Table 2, distribution of *BRCA2 *mutation carriers and non-carriers was quite identical in the different subsets of patients according to such pathological parameters (thus, no statistically significant correlation was observed – not shown).

**Table 1:** Distribution of patients according to *BRCA2 *mutation status and age of onset

Age Class	*BRCA2 *mutation negative	*BRCA2 *mutation positive	Total
*20*	1		1
*25*	2	1	3
*30*	21	2	23
*35*	27	3	30
*40*	38	7	45
*45*	54	6	60
*50*	45	4	49
*55*	57	8	65
*60*	47	3	50
*65*	50	5	55
*70*	56	1	57
*75*	36	2	38
*80*	21	1	22
*85*	9	1	10

	**464**	**44**	**508**

Taking into consideration the primary tumour morphology, 348 (68%) ductal carcinomas, 62 (12%) lobular carcinomas, and 102 (20%) other histological types were registered. The *BRCA2 *mutations were more prevalent in lobular (7/62; 11%) than in ductal carcinomas (20/348; 6%); again, differences were not statistically significant.

**Figure 1:**
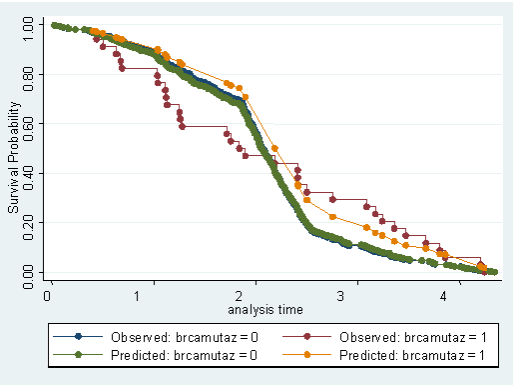
**Overall survival curves based on the Kaplan-Meier method**. Comparison between observed and predicted survival data for each subset of patients (with or without *BRCA2 *mutations) is reported.

**Figure 2:**
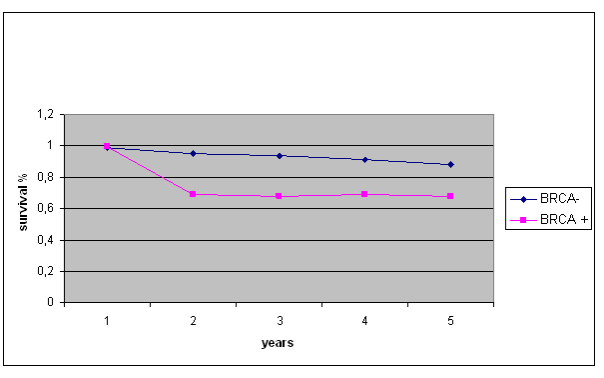
**Relative five-year survival for breast cancer patients with or without *BRCA2 *mutations (BRCA+/-), adjusted by age according to Brenner**.

**Figure 3:**
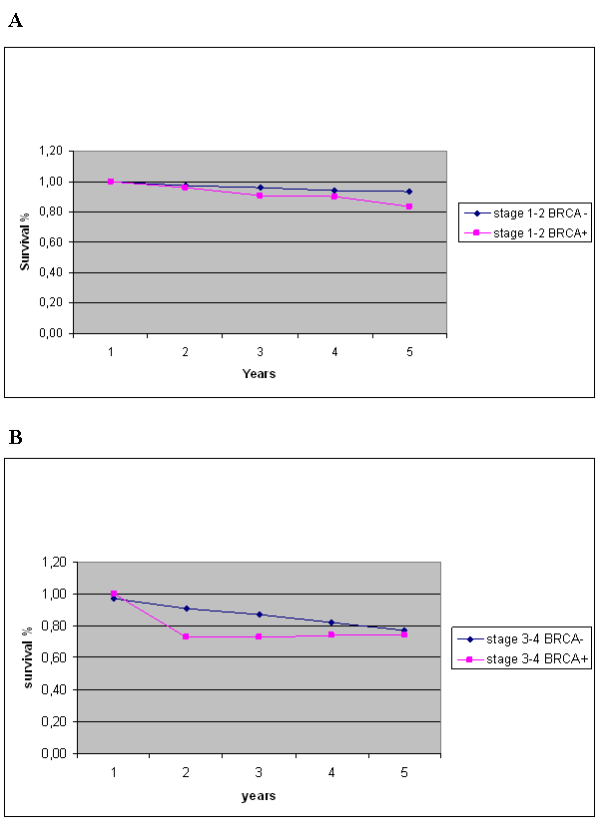
**Relative five-year survival for breast cancer patients according to (A) localized disease (stage 1–2) or (B) metastatic disease (stage 3–4), and presence or absence of *BRCA2 *mutations (BRCA+/-)**.

The five-year survival rate was 81% (80%, adjusted by age) among *BRCA2 *mutation carriers and 91% (92%, adjusted by age) among patients negative for *BRCA2 *mutations. Overall, the five-year relative survival rate for breast cancer cases from our series was 85%. Evaluation of the overall survival curves using the Kaplan-Meier method indicated that patients carrying *BRCA2 *mutations presented a lower breast cancer-specific survival in comparison with those resulted negative for *BRCA2 *mutations, within the first two years from diagnosis (Figure 1). Considering the entire observation period of five years from diagnosis, the survival curves tended to merge with no significant difference in outcome between the two groups (Figure 1). Furthermore, no difference in survival among familial and sporadic *BRCA2 *mutated cases was observed (not shown). As estimated by Cox regression analysis, the hazard ratio of patients positive for *BRCA2 *mutations was found to be 0.7 (95% CI, 0.46–1.37), after adjustment by age (Figure 2), and about 0.8 (95% CI, 0.48–1.62), after adjustment by disease stage (Figure 3). Hazard ratios were quite identical for both subsets when adjusted for tumour grade (0.82; 95% CI, 0.53–0.98) and receptor status (0.85; 95% CI, 0.64–0.97) (not shown).

Finally, multivariate Cox regression analysis was performed considering all variables (pT, pN, M, ER, PR, and *BRCA2 *mutations). The presence of metastases was the only parameter with a significant impact on prognosis (p < 0.001; hazard ratio, 8.939; 95% CI, 4.68–17.1). However, such a prognostic factor was not able to exert a confounding effect on mutation-based survival curves due to the low number of patients with distant metastases (33/508; 6%), who even showed a similar prevalence of *BRCA2 *mutation-negative (30/464; 6%) and mutation-positive (3/44; 7%) cases (see Table 2). No other association between *BRCA2 *mutation status and overall survival was observed for the remaining variables.

**Table 2:** Distribution of *BRCA2 *cases according to the TNM and receptor status

	Primary tumour size		
*BRCA2 *mutation	T1-2	T3-4	Total
**Negative**	421 *91%*	43 *9%*	464
**Positive**	41 *93%*	3 *7%*	44
**Total**	462 *91%*	46 *9%*	508
			
	**Lymph node metastasis**		
***BRCA2 *mutation**	**Negative**	**Positive**	**Total**
**Negative**	263 *57%*	201 *43%*	464
**Positive**	24 *55%*	20 *45%*	44
**Total**	287 *56%*	221 *44%*	508
			
	**Distant metastasis**		
***BRCA2 *mutation**	**Absent**	**Present**	**Total**
**Negative**	434 *94%*	30 *6%*	464
**Positive**	41 *93%*	3 *7%*	44
**Total**	475 *94%*	33 *6%*	508
			
	**Estrogen receptor**		
***BRCA2 *mutation**	**Negative**	**Positive**	**Total**
**Negative**	91 *26%*	257 *74%*	348
**Positive**	5 *29%*	12 *71%*	17
**Total**	96 *26%*	269 *74%*	365
			
	**Progesterone receptor**		
***BRCA2 *mutation**	**Negative**	**Positive**	**Total**
**Negative**	150 *44%*	189 *56%*	339
**Positive**	6 *46%*	7 *54%*	13
**Total**	156 *44%*	196 *56%*	352

## Discussion and conclusion

Breast cancers carrying *BRCA1 *or *BRCA2 *germline mutations often occur in younger women as well as present a high tumour grade and/or lack of expression of estrogen/progesterone receptors (mostly, among *BRCA1*-positive tumours) [11-16,30-32]. Although these features have been associated with a poor prognosis, the relationship between the occurrence of a *BRCA1 *or *BRCA2 *mutation and the effect on overall survival is still controversial [17-22,31,33-35].

In this study, we tried to clarify the role of *BRCA *mutations on the outcome of breast cancer patients from North Sardinia, where an official cancer registry is available [26]. In particular, we evaluated the 5-year survival rates among women who had received the diagnosis of breast cancer from 1997 to 2002 and gave their consent to undergo a *BRCA *genetic testing. Among the 508 analyzed patients, we assessed the breast cancer-specific survival rates for women with (44 cases; 9%) or without (464 cases; 91%) a *BRCA2 *germline mutation.

Using the Kaplan-Meier method, the survival rate of patients with a positive *BRCA2 *genetic test was lower than that of patients with negative genetic tests within the first two years after diagnosis in our series. However, the two survival curves tended to merge at the end of five years from diagnosis (see Figure 1). This trend may indeed account for the absence of significance of the Cox regression for *BRCA2 *status, due to the failure of proportionality of hazards (a situation in which calculation of a total hazard ratio with the Cox model is unsuitable). The relative survival of the entire Sardinian series at five years from diagnosis is slightly below the average survival rate observed in breast cancer cases from the other Italian regions [17]; moreover, it always remains lower in the subset of *BRCA2 *mutation carriers than in that of *BRCA2 *mutation-negative patients, regardless the adjustments according to the different prognostic parameters in multivariate analysis (see Figures 2 and 3). Indeed, the relative survival seems to be worse in cases with positive *BRCA2 *genetic tests, even after adjustment for age of onset (which represents one of the factors with the greatest influence on cancer prognosis, according to the studies in other populations [34-36]). Although survival is also deeply influenced by disease stage at the time of diagnosis, we observed no significant difference between the two *BRCA2 *subsets after stratification by stage.

Although our study has a number of limitations mainly due to the fact that we identified only a limited fraction (44/508; 9%) of mutation carriers and, thus, the subgroup analyses relied on a small number of subjects, we can conclude that a prolonged follow-up observation seems to minimize the effect of the presence of *BRCA2 *germline mutations on prognosis among breast cancer patients from Sardinian population.

Our findings are consistent with data recently reported in Israeli women of Ashkenazi Jewish ancestry (who present a high prevalence of hereditary breast cancer and *BRCA *founder mutations) [22] as well as in a Dutch series of *BRCA1*-associated breast carcinoma patients [37]. In both studies, the breast cancer-specific survivals for carriers and noncarriers of *BRCA *mutations were similar, even considering a longer period of observation (ten years) from diagnosis [22,37]. On the basis of these results, one could speculate that additional factors may influence the prognosis in such patients.

Search for prognostic factors in breast cancer patients is still a challenge; several lifestyle and environmental risk factors for breast cancer are being investigated. For example, evidence have been recently found that an increase in body mass index is associated with a poorer prognosis in women receiving diagnosis of breast cancer [36]. Probably, further studies toward the comprehension of the underlying interactions between all genetic and environmental factors could really improve the classification of the different subsets of patients who would be expected to have better or worse prognosis as well as to be more or less likely to respond to specific therapeutic interventions.

## Competing interests

The authors declare that they have no competing interests.

## Authors' contributions

MB participated to the design of the study and performed statistical analyses. RC participated to analysis and interpretation of data. VC participated to statistical analysis. OS participated to analysis and interpretation of data. DP performed the data management. AC participated to patients' collection and performed some screening analyses. FT participated to patients' collection. MP participated to mutation analysis. GrP performed mutation screening. GiP conceived of the study and drafted the manuscript. All authors read and approved the final manuscript.

## Pre-publication history

The pre-publication history for this paper can be accessed here:

http://www.biomedcentral.com/1471-2407/9/62/prepub

## References

[B1] Lønning PE Breast cancer prognostication and prediction: are we making progress? Ann Oncol 2007 18 Suppl 8 viii3 7 10.1093/annonc/mdm260 17890212

[B2] van't Veer LJ Dai H Vijver MJ van de He YD Hart AA Mao M Peterse HL Kooy K van der Marton MJ Witteveen AT Schreiber GJ Kerkhoven RM Roberts C Linsley PS Bernards R Friend SH Gene expression profiling predicts clinical outcome of breast cancer Nature 2002 415 530 536 10.1038/415530a 11823860

[B3] Jarzabek K Koda M Kozlowski L Mittre H Sulkowski S Kottler ML Wolczynski S Distinct mRNA, protein expression patterns and distribution of oestrogen receptors alpha and beta in human primary breast cancer: correlation with proliferation marker Ki-67 and clinicopathological factors Eur J Cancer 2005 41 2924 34 10.1016/j.ejca.2005.09.010 16289616

[B4] Ford D Easton DF Bishop DT Narod SA Godgar DE Risks of cancer in BRCA1-mutation carriers. Breast Cancer Linkage Consortium Lancet 1994 343 692 695 10.1016/S0140-6736(94)91578-4 7907678

[B5] Easton DF Ford D Bishop DT Breast Cancer Linkage Consortium. Breast and ovarian cancer incidence in *BRCA1*-mutation carriers Am J Hum Genet 1995 56 265 271 7825587 PMC1801337

[B6] Easton DF Steele L Fields P Ormiston W Averill D Daly PA McManus R Neuhausen SL Ford D Wooster R Cannon-Albright LA Stratton MR Goldgar DE Cancer risks in two large breast cancer families linked to BRCA2 on chromosome 13q12-13 Am J Hum Genet 1997 61 120 128 9245992 10.1086/513891 PMC1715847

[B7] Ford D Easton DF Stratton M Narod S Goldgar D Devilee P Bishop DT Weber B Lenoir G Chang-Claude J Sobol H Teare MD Struewing J Arason A Scherneck S Peto J Rebbeck TR Tonin P Neuhausen S Barkardottir R Eyfjord J Lynch H Ponder BA Gayther SA Zelada-Hedman M Genetic heterogeneity and penetrance analysis of the BRCA1 and BRCA2 genes in breast cancer families. The Breast Cancer Linkage Consortium Am J Hum Genet 1998 62 676 689 9497246 10.1086/301749 PMC1376944

[B8] Kirova YM Stoppa-Lyonnet D Savignoni A Sigal-Zafrani B Fabre N Fourquet A Institut Curie Breast Cancer Study Group. Risk of breast cancer recurrence and contralateral breast cancer in relation to BRCA1 and BRCA2 mutation status following breast-conserving surgery and radiotherapy Eur J Cancer 2005 41 2304 11 10.1016/j.ejca.2005.02.037 16140006

[B9] Dent R Warner E Screening for hereditary breast cancer Semin Oncol 2007 34 392 400 10.1053/j.seminoncol.2007.07.002 17920893

[B10] Metcalfe KA Lubinski J Ghadirian P Lynch H Kim-Sing C Friedman E Foulkes WD Domchek S Ainsworth P Isaacs C Tung N Gronwald J Cummings S Wagner T Manoukian S Møller P Weitzel J Sun P Narod SA Hereditary Breast Cancer Clinical Study Group. Predictors of contralateral prophylactic mastectomy in women with a BRCA1 or BRCA2 mutation: the Hereditary Breast Cancer Clinical Study Group J Clin Oncol 2008 26 1093 7 10.1200/JCO.2007.12.6078 18195327

[B11] Adem C Reynolds C Soderberg CL Slezak JM McDonnell SK Sebo TJ Schaid DJ Myers JL Sellers TA Hartmann LC Jenkins RB Pathologic characteristics of breast parenchyma in patients with hereditary breast carcinoma, including BRCA1 and BRCA2 mutation carriers Cancer 2003 97 1 11 10.1002/cncr.11048 12491499

[B12] Chappuis PO Nethercot V Foules WD Clinico-pathological characteristics of BRCA1 and BRCA2-related breast cancer Semin Surg Oncol 2000 18 287 295 10.1002/(SICI)1098-2388(200006)18:4<287::AID-SSU3>3.0.CO;2-5 10805950

[B13] Honrado E Benítez J Palacios J Histopathology of BRCA1- and BRCA2-associated breast cancer Crit Rev Oncol Hematol 2006 59 27 39 10.1016/j.critrevonc.2006.01.006 16530420

[B14] Palacios J Honrado E Osorio A Cazorla A Sarrió D Barroso A Rodríguez S Cigudosa JC Diez O Alonso C Lerma E Sánchez L Rivas C Benítez J Immunohistochemical characteristics defined by tissue microarray of hereditary breast cancer not attributable to BRCA1 or BRCA2 mutations: differences from breast carcinomas arising in BRCA1 and BRCA2 mutation carriers Clin Cancer Res 2003 9 3606 3614 14506147

[B15] Lakhani SR Jacquemier J Sloane JP Gusterson BA Anderson TJ Vijver MJ van de Farid LM Venter D Antoniou A Storfer-Isser A Smyth E Steel CM Haites N Scott RJ Goldgar D Neuhausen S Daly PA Ormiston W McManus R Scherneck S Ponder BA Ford D Peto J Stoppa-Lyonnet D Bignon YJ Struewing JP Spurr NK Bishop DT Klijn JG Devilee P Cornelisse CJ Lasset C Lenoir G Barkardottir RB Egilsson V Hamann U Chang-Claude J Sobol H Weber B Stratton MR Easton DF Multifactorial analysis of differences between sporadic breast cancers and cancers involving BRCA1 and BRCA2 mutations J Natl Cancer Inst 1998 90 1138 1145 10.1093/jnci/90.15.1138 9701363

[B16] The Breast Cancer Linkage Consortium. Pathology of familial breast cancer: differences between breast cancers in carriers of BRCA1 or BRCA2 mutations and sporadic cases Lancet 1997 349 1505 1510 10.1016/S0140-6736(96)10109-4 9167459

[B17] Foulkes WD Chappuis PO Wong N Brunet JS Vesprini D Rozen F Yuan ZQ Pollak MN Kuperstein G Narod SA Begin LR Primary node negative breast cancer in BRCA1 mutation carriers has a poor outcome Ann Oncol 2000 11 307 13 10.1023/A:1008340723974 10811497

[B18] Robson ME Chappuis PO Satagopan J Wong N Boyd J Goffin JR Hudis C Roberge D Norton L Begin LR Offit K Foulkes WD A combined analysis of outcome following breast cancer: differences in survival based on BRCA1/BRCA2 mutation status and administration of adjuvant treatment Breast Cancer Res 2004 6 14680495 10.1186/bcr658 PMC314444

[B19] Stoppa-Lyonnet D Ansquer Y Dreyfus H Gautier C Gauthier-Villars M Bourstyn E Clough KB Magdelenat H Pouillart P Vincent-Salomon A Fourquet A Asselain B Familial invasive breast cancers: worse outcome related to BRCA1 mutations J Clin Oncol 2000 18 4053 4059 11118466 10.1200/JCO.2000.18.24.4053

[B20] Johannsson OT Ranstam J Borg A Olsson H Survival of BRCA1 breast and ovarian cancer patients: a population-based study from southern Sweden J Clin Oncol 1998 16 397 404 9469321 10.1200/JCO.1998.16.2.397

[B21] Brekelmans CTM Seynaeve C Menke-Pluymers M Survival and prognostic factors in BRCA1-associated breast cancer Ann Oncol 2006 17 391 400 10.1093/annonc/mdj095 16322115

[B22] Rennert G Bisland-Naggan S Barnett-Griness O Clinical outcomes of breast cancer in carriers of BRCA1 and BRCA2 mutations N Engl J Med 2007 357 115 123 10.1056/NEJMoa070608 17625123

[B23] Palmieri G Palomba G Cossu A Pisano M Dedola MF Sarobba M Farris A Olmeo N Contu A Pasca A Satta M Persico I Carboni A Cossu-Rocca P Contini M Mangion J Stratton MR Tanda F BRCA1 and BRCA2 germline mutations in Sardinian breast cancer families and their implications for genetic counseling Ann Oncol 2002 13 1899 1907 10.1093/annonc/mdf326 12453858

[B24] Palomba G Pisano M Cossu A Budroni M Dedola MF Farris A Contu A Baldinu P Tanda F Palmieri G Spectrum and prevalence of *BRCA1 *and *BRCA2 *germline mutations in Sardinian breast cancer patients through a hospital-based screening Cancer 2005 104 1172 1179 10.1002/cncr.21298 16047344

[B25] Palomba G Cossu A Friedman E Budroni M Farris A Contu A Pisano M Baldinu P Sini MC Tanda F Palmieri G Origin and distribution of the BRCA2-8765delAG mutation in breast cancer BMC Cancer 2007 7 132 17640379 10.1186/1471-2407-7-132 PMC1940259

[B26] Budroni M Cesaraccio R Pirino D Sechi O Oggiano M Piras D Sechi A Cossu A Palmieri G Tanda F Curado MP, Edwards B, Shin HR, Storm H, Ferlay J, Heanue M, Boyle P Cancer incidence in Sassari Province (1998–2002) Cancer Incidence in Five Continents, International Agency for Research on Cancer (IARC) Scientific Publications, No. 160 2007 IX Lyon, IARC

[B27] Hakulinen T Cancer survival corrected for heterogeneity in patient withdrawal Biometrics 1982 38 933 942 10.2307/2529873 7168796

[B28] Verdecchia A Capocaccia R Hakulinen T Berrino F, SAnt M, Verdecchia A, Capocaccia R, Hakulinen T, Esteve J Methods of data analysis Survival of cancer patients in Europe. The EUROCARE study. IARC scientific publication 95 1995 Lyon International Agency for Research on Cancer

[B29] Brenner H Hakulinen T Age adjustment of cancer survival rates: methods, point estimates and standard errors Br J Cancer 2005 93 372 5 10.1038/sj.bjc.6602704 16052220 PMC2361559

[B30] Mote PA Leary JA Avery KA Sandelin K Chenevix-Trench G Kirk JA Clarke CL Germ-line mutations in BRCA1 or BRCA2 in the normal breast are associated with altered expression of estrogen-responsive proteins and the predominance of progesterone receptor Genes Chromosomes Cancer 2004 39 236 48 10.1002/gcc.10321 14732925

[B31] Veronesi A de Giacomi C Magri MD Lombardi D Zanetti M Scuderi C Dolcetti R Viel A Crivellari D Bidoli E Boiocchi M Familial breast cancer: characteristics and outcome of BRCA 1–2 positive and negative cases BMC Cancer 2005 5 70 15996267 10.1186/1471-2407-5-70 PMC1184063

[B32] Musolino A Bella MA Bortesi B Michiara M Naldi N Zanelli P Capelletti M Pezzuolo D Camisa R Savi M Neri TM Ardizzoni A BRCA mutations, molecular markers, and clinical variables in early-onset breast cancer: a population-based study Breast 2007 16 280 92 10.1016/j.breast.2006.12.003 17257844

[B33] Eerola H Vahteristo P Sarantaus L Survival of breast cancer patients in *BRCA1, BRCA2*, and non-*BRCA1*/2 breast cancer families: a relative survival analysis from Finland Int J Cancer 2001 93 368 72 10.1002/ijc.1341 11433401

[B34] El-Tamer M Russo D Troxel A Survival and recurrence after breast cancer in *BRCA1*/2 mutation carriers Ann Surg Oncol 2004 11 157 64 10.1245/ASO.2004.05.018 14761918

[B35] Moller P Evans DG Reis MM Gregory H Anderson E Maehle L Lalloo F Howell A Apold J Clark N Lucassen A Steel CM Surveillance for familial breast cancer: Differences in outcome according to BRCA mutation status Int J Cancer 2007 121 1017 1020 10.1002/ijc.22789 17471561

[B36] Barnett GC Shah M Redman K Easton DF Ponder BAJ Pharoah PDP Risk factors for the incidence of breast cancer: do they affect survival from the disease? J Clin Oncol 2008 26 3310 3316 10.1200/JCO.2006.10.3168 18612147

[B37] Brekelmans CT Seynaeve C Menke-Pluymers M Brüggenwirth HT Tilanus-Linthorst MM Bartels CC Kriege M van Geel AN Crepin CM Blom JC Meijers-Heijboer H Klijn JG Survival and prognostic factors in BRCA1-associated breast cancer Ann Oncol 2006 17 391 400 10.1093/annonc/mdj095 16322115

